# First report on the identification and characterization of mammalian orthoreovirus from sheep in China

**DOI:** 10.1128/spectrum.00847-24

**Published:** 2024-10-15

**Authors:** Dengshuai Zhao, Ping Li, Yuanhang Zhang, Dixi Yu, Tianyu Wang, Keshan Zhang

**Affiliations:** 1 Guangdong Provincial Key Laboratory of Animal Molecular Design and Precise Breeding, College of Animal Science and Technology, Foshan University, Foshan, Guangdong Province, China; 2 College of Veterinary Medicine, Henan Agricultural University, Zhengzhou, China; Thomas Jefferson University, Philadelphia, Pennsylvania, USA

**Keywords:** mammalian orthoreovirus, sheep, whole genome sequencing, phylogenetic analysis

## LETTER

Mammalian orthoreovirus (MRV) infects various mammalian species worldwide, including humans, pigs, bats, cattle, ferrets, dogs, cats, and civets (1–9). Various reports of clinical cases of MRV in humans and other mammalian hosts have presented evidence confirming the possibility of interspecies transmission (10, 11). MRV typically presents as asymptomatic or mild respiratory and gastrointestinal infections (12). MRV is capable of inducing neurological symptoms. However, cases with neurological symptoms are less reported than those with gastrointestinal and respiratory symptoms. Infection with MRV types 2 and 3 may result in necrotizing encephalopathy and meningitis (13, 14), whereas MRV types 1 and 3 may infect the central nervous system of mice via various pathways (15, 16). Thus, MRV poses a risk of interspecies transmission and can potentially cause severe clinical symptoms.

Here, we report the first identification of MRV from sheep in China. The sheep were located in high-altitude regions and showed clinical symptoms of watery diarrhea and dehydration. We collected multiple serum and fecal samples as well as anal swabs and tissue specimens from various segments of the gastrointestinal tract, including the duodenum, jejunum, ileum, and cecum. Agarose gel electrophoresis revealed the presence of MRV in the samples. Further analysis using whole genome sequencing revealed that the samples were MRV positive. We utilized high-throughput sequencing technology to obtain the complete genomic sequence of the MRV strain (GR/2023) and determined the lengths of 10 genomic segments, along with the proteins they potentially encode (GenBank accession nos. OR902351–OR902360) as follows: *L1*: 3,864 bp (λ3: 1,287 aa), *L2*: 3,870 bp (λ2: 1,289 aa), *L3*: 3,840 bp (λ1: 1,279 aa), *M1*: 2,244 bp (μ2: 747 aa), *M2*: 2,127 bp (μ1: 708 aa), *M3*: 2,166 (μNS: 721 aa), *S1*: 1,425 bp (σ1: 474 aa and σ1s: 147 aa), *S2*: 1,323 bp (σ2: 440 aa), *S3*: 1,161 bp (σNS: 386 aa), and *S4*: 1,176 bp (σ3: 391 aa).

**Fig 1:**
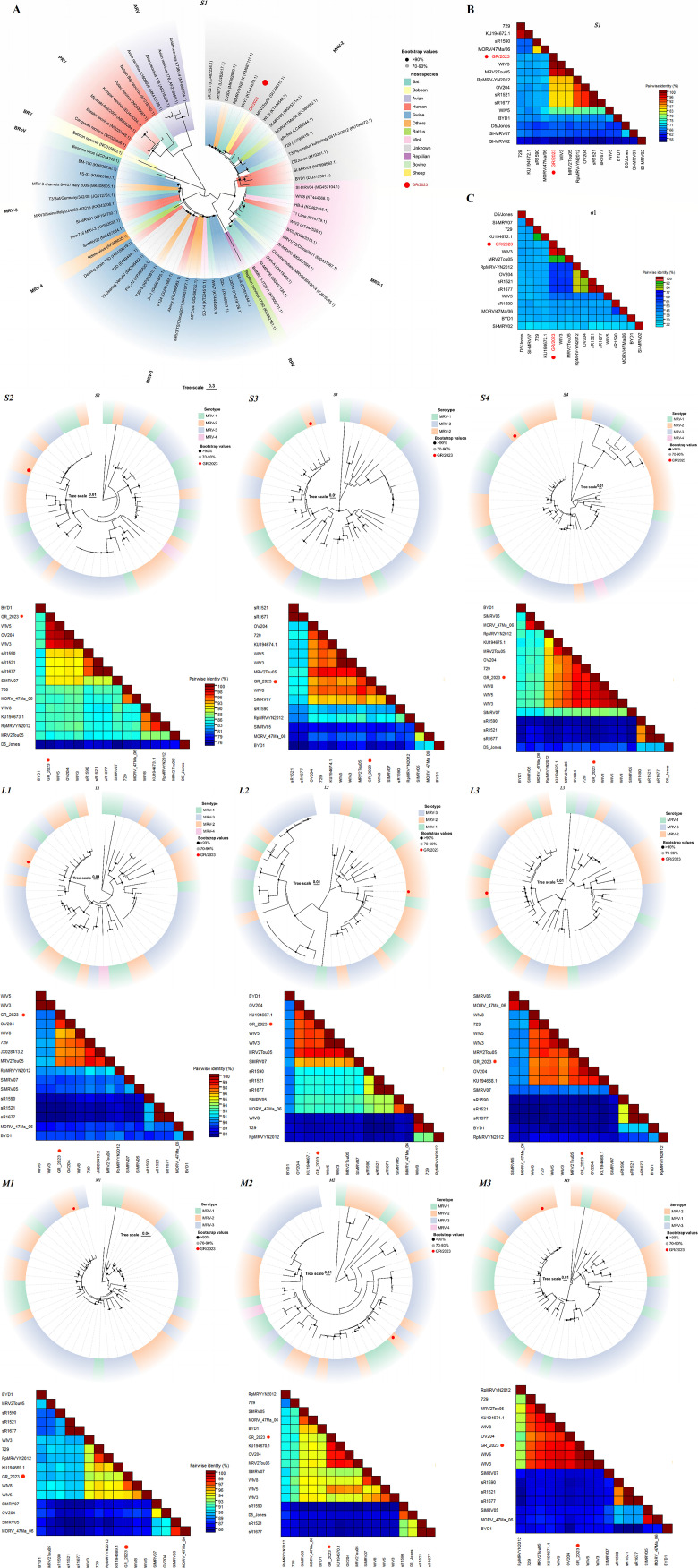
Phylogenetic analysis of GR/2023. (**A)** Phylogenetic tree based on the *S1* genomic segment of GR/2023 and the most relevant coding sequences of other positive-sense reovirus strains in GenBank. The tree was constructed using the neighbor-joining method in MEGA software with 1,000 bootstrap replicates. Different MRV lineages and virus hosts are marked with different color blocks, and GR/2023 is represented by a red circle. (**B)** Heatmap of the pairwise genetic distance between the coding sequence of the GR/2023 *S1* fragment and other MRV2 strains was constructed using Sequence Demarcation Tool Version 1.2 (SDTv1.2) software. The red circle represents GR/2023. (**C)** Paired genetic distance heatmap based on the amino acid sequence of σ1, GR/2023, and other MRV2 strains using SDTv1.2 software. The red circle represents GR/2023. *S2–S4*, *L1–L3*, and *M1–M3* phylogenetic analysis and pairwise genetic distance heatmap of GR/2023 based on the complete nucleotide coding sequences of *S2–S4*, *L1–L3*, and *M1–M3* segments. Different MRV lineages are marked with different color blocks. The red circle represents GR/2023. The phylogenetic tree was constructed in MEGA software using the neighbor-joining method. The evolutionary tree was beautified using tvBOT (19). The pairwise genetic distance heatmap was constructed using SDTv1.2 software.

The σ1 protein, encoded by the *S1* segment, is crucial for cellular attachment, type-specific serum neutralization, and hemagglutinin activity. Furthermore, σ1 protein variations are used to classify MRVs into four serotypes: type 1 (MRV1) Lang, type 2 (MRV2) Jones, type 3 (MRV3) Dearing, and putative type 4 (MRV4) Ndelle (17, 18). We further compared the nucleotide sequence of GR/2023 with those of other MRVs from various hosts and serotypes, revealing a similarity of 97.15%–99.65% with MRVs from different hosts and serotypes. Among them, GR/2023 exhibited the highest nucleotide sequence similarity (99.65%) with the bat-derived MRV strain WIV3 (KT444578). A phylogenetic analysis was conducted based on the *S1* gene (Fig. 1A). The findings indicated that GR/2023 clustered within the MRV2 lineage and exhibited the closest genetic relationship with WIV3. Similarly, the pairwise genetic distance heat map, derived from the coding sequence of the *S1* gene fragment and the inferred amino acid sequence, demonstrated that GR/2023 shared the highest similarity with WIV3 (Fig. 1B and C). These findings suggest that GR/2023 may have undergone recombination events with WIV3 and other strains. Although phylogenetic analysis based on the *S1* gene indicated that GR/2023 belonged to MRV2, analyses based on other genes revealed lower levels of homology between GR/2023 and MRV2 (Fig. 1 *S2*–*S4*, *L1*–*L3*, and *M1*–*M3*). Previous analyses of nucleotide sequence similarity revealed that *L1* and *L3* exhibited the highest similarity with MRV3 strain SD-14 (98.21%) and MRV1 strain WIV2 (98.92%), derived from *Mink* and *Myotis ricketti*. Pairwise genetic distance analysis based on the amino acid sequences encoded by *L1* and *L3* also showed lower similarity with MRV2 strains compared with other gene coding sequences (Fig. 1 L1, L3). In our study, GR/2023 *L2* and *S2* showed the highest similarity with the human-derived MRV2 Tou05 strain (97.15%) and Osaka2005 strain (98.94%), respectively, providing further evidence indicating that GR/2023 originated from the recombination of WIV3 with other strains from diverse origins. This discovery is of paramount importance for further exploration of the epidemiologic and molecular characteristics of ovine-origin MRVs, as well as understanding the diversity of MRV hosts and recombination events. Additional research focusing on the development of a diagnostic kit and an effective vaccine is crucial for developing effective control and prevention strategies as well as protecting the health and productivity of sheep herds in China.

## Supplementary Material

Reviewer comments

## Data Availability

Sequences have been submitted to GenBank under accession numbers OR902351–OR902360.

## SUPPLEMENTAL MATERIAL

OPEN PEER REVIEW reviewer-comments.pdf An accounting of the reviewer comments and feedback.

ASM does not own the copyrights to Supplemental Material that may be linked to, or accessed through, an article. The authors have granted ASM a non-exclusive, world-wide license to publish the Supplemental Material files. Please contact the corresponding author directly for reuse.

## References

[1] Lelli D, Moreno A, Lavazza A, Bresaola M, Canelli E, Boniotti MB, Cordioli P. 2013. Identification of mammalian orthoreovirus type 3 in Italian bats. Zoonoses Public Health 60:84–92. doi:10.1111/zph.12001 22931153

[2] Qin P, Li H, Wang JW, Wang B, Xie RH, Xu H, Zhao LY, Li L, Pan Y, Song Y, Huang YW. 2017. Genetic and pathogenic characterization of a novel reassortant mammalian orthoreovirus 3 (MRV3) from a diarrheic piglet and seroepidemiological survey of MRV3 in diarrheic pigs from east China. Vet Microbiol 208:126–136. doi:10.1016/j.vetmic.2017.07.021 28888627 PMC7117289

[3] Yang XL, Tan B, Wang B, Li W, Wang N, Luo CM, Wang MN, Zhang W, Li B, Peng C, Ge XY, Zhang LB, Shi ZL. 2015. Isolation and identification of bat viruses closely related to human, porcine and mink orthoreoviruses. J Gen Virol 96:3525–3531. doi:10.1099/jgv.0.000314 26475793 PMC7081072

[4] Li Z, Shao Y, Liu C, Liu D, Guo D, Qiu Z, Tian J, Zhang X, Liu S, Qu L. 2015. Isolation and pathogenicity of the mammalian orthoreovirus MPC/04 from masked civet cats. Infect Genet Evol 36:55–61. doi:10.1016/j.meegid.2015.08.037 26325682 PMC7106251

[5] Decaro N, Campolo M, Desario C, Ricci D, Camero M, Lorusso E, Elia G, Lavazza A, Martella V, Buonavoglia C. 2005. Virological and molecular characterization of a mammalian orthoreovirus type 3 strain isolated from a dog in Italy. Vet Microbiol 109:19–27. doi:10.1016/j.vetmic.2005.05.014 15964158 PMC7125552

[6] Steyer A, Gutiérrez-Aguire I, Kolenc M, Koren S, Kutnjak D, Pokorn M, Poljšak-Prijatelj M, Racki N, Ravnikar M, Sagadin M, Fratnik Steyer A, Toplak N. 2013. High similarity of novel orthoreovirus detected in a child hospitalized with acute gastroenteritis to mammalian orthoreoviruses found in bats in Europe. J Clin Microbiol 51:3818–3825. doi:10.1128/JCM.01531-13 24025904 PMC3889772

[7] Kohl C, Lesnik R, Brinkmann A, Ebinger A, Radonić A, Nitsche A, Mühldorfer K, Wibbelt G, Kurth A. 2012. Isolation and characterization of three mammalian orthoreoviruses from European bats. PLoS One 7:e43106. doi:10.1371/journal.pone.0043106 22905211 PMC3419194

[8] Anbalagan S, Spaans T, Hause BM. 2014. Genome sequence of the novel reassortant mammalian orthoreovirus strain MRV00304/13, Isolated from a calf with diarrhea from the United States. Genome Announc 2:e00451-14. doi:10.1128/genomeA.00451-14 24874671 PMC4038876

[9] Lian H, Liu Y, Zhang S, Zhang F, Hu R. 2013. Novel orthoreovirus from mink, China, 2011. Emerg Infect Dis 19:1985–1988. doi:10.3201/eid1912.130043 24274037 PMC3840883

[10] Chua KB, Voon K, Crameri G, Tan HS, Rosli J, McEachern JA, Suluraju S, Yu M, Wang LF. 2008. Identification and characterization of a new orthoreovirus from patients with acute respiratory infections. PLoS One 3:e3803. doi:10.1371/journal.pone.0003803 19030226 PMC2583042

[11] Chua KB, Voon K, Yu M, Keniscope C, Abdul Rasid K, Wang LF. 2011. Investigation of a potential zoonotic transmission of orthoreovirus associated with acute influenza-like illness in an adult patient. PLoS One 6:e25434. doi:10.1371/journal.pone.0025434 22022394 PMC3192755

[12] Schiff LA, Nibert ML, Tyler KL. 2007. Orthoreoviruses and their replication

[13] Ouattara LA, Barin F, Barthez MA, Bonnaud B, Roingeard P, Goudeau A, Castelnau P, Vernet G, Paranhos-Baccalà G, Komurian-Pradel F. 2011. Novel human reovirus isolated from children with acute necrotizing encephalopathy. Emerg Infect Dis 17:1436–1444. doi:10.3201/eid1708.101528 21801621 PMC3381585

[14] Tyler KL, Barton ES, Ibach ML, Robinson C, Campbell JA, O’Donnell SM, Valyi-Nagy T, Clarke P, Wetzel JD, Dermody TS. 2004. Isolation and molecular characterization of a novel type 3 reovirus from a child with meningitis. J Infect Dis 189:1664–1675. doi:10.1086/383129 15116303

[15] Spriggs DR, Fields BN. 1982. Attenuated reovirus type 3 strains generated by selection of haemagglutinin antigenic variants. Nature 297:68–70. doi:10.1038/297068a0 6175910

[16] Weiner HL, Drayna D, Averill DR, Fields BN. 1977. Molecular basis of reovirus virulence: role of the S1 gene. Proc Natl Acad Sci U S A 74:5744–5748. doi:10.1073/pnas.74.12.5744 271999 PMC431870

[17] Lelli D, Beato MS, Cavicchio L, Lavazza A, Chiapponi C, Leopardi S, Baioni L, De Benedictis P, Moreno A. 2016. First identification of mammalian orthoreovirus type 3 in diarrheic pigs in Europe. Virol J 13:139. doi:10.1186/s12985-016-0593-4 27519739 PMC4983005

[18] Attoui H, Biagini P, Stirling J, Mertens PP, Cantaloube JF, Meyer A, de Micco P, de Lamballerie X. 2001. Sequence characterization of Ndelle virus genome segments 1, 5, 7, 8, and 10: evidence for reassignment to the genus orthoreovirus, family reoviridae. Biochem Biophys Res Commun 287:583–588. doi:10.1006/bbrc.2001.5612 11554769

[19] Xie J, Chen Y, Cai G, Cai R, Hu Z, Wang H. 2023. Tree visualization by one table (tvBOT): a web application for visualizing, modifying and annotating phylogenetic trees. Nucleic Acids Res 51:W587–W592. doi:10.1093/nar/gkad359 37144476 PMC10320113

